# A7 THERAPEUTIC DIETARY INTERVENTION IN ADULT CROHN’S DISEASE PATIENTS: EFFECTS ON REMISSION MARKERS, QUALITY OF LIFE, AND PHYSICAL ACTIVITY

**DOI:** 10.1093/jcag/gwae059.007

**Published:** 2025-02-10

**Authors:** B R Toews, C Lu, M Raman, R Reimer

**Affiliations:** University of Calgary Faculty of Kinesiology, Calgary, AB, Canada; Division of Gastroenterology and Hepatology, Department of Medicine, University of Calgary, Calgary, AB, Canada; Division of Gastroenterology and Hepatology, Department of Medicine, University of Calgary, Calgary, AB, Canada; Department of Biochemistry and Molecular Biology, Cumming School of Medicine, Calgary, AB, Canada

## Abstract

**Background:**

Current evidence recognizes diet and physical activity as important factors in influencing the natural history of Crohn’s disease (CD); however, the relationship between dietary components and disease relapse remains unclear. Patients with active CD have a lower quality of life (QoL) compared to healthy controls.

**Aims:**

The aims of this study were threefold: 1) to explore the effect of a CD specific therapeutic dietary intervention (CD-TDI) upon clinical and biochemical remission in adults with active, mild luminal CD, 2) to investigate outcomes related to patient-reported health QoL, and 3) to investigate the relationship between IBD parameters and physical activity. We hypothesized that a CD-TDI would be more effective when compared to conventional management (CM) to induce clinical and biochemical remission and improve patient-reported QoL in symptomatic and asymptomatic patients with active, mild luminal CD.

**Methods:**

This was a randomized controlled trial whereby 51 participants were randomly allocated in a 2:1 ratio to the intervention group (CD-TDI) or CM for 13 weeks. Patients receiving the CD-TDI, an anti-inflammatory diet developed specifically for CD with foundational principles rooted in the Mediterranean diet, were offered patient-centered counseling by a Registered Dietitian for 12 weeks. QoL was measured using the 12-item short form health survey (SF-12). Physical activity was measured using ActivPAL® inclinometers (PAL Technologies, Glasgow). The Harvey Bradshaw Index (HBI) and fecal calprotectin (mcg/g) were used to assess clinical and biochemical remission.

**Results:**

The CD-TDI group experienced both significant decreases in HBI from baseline to week 13 (Wk0: 3.0, IQR:1.0-5.3; Wk13: 2.0, IQR:0-3.0; p=0.05) and FCP from 130 ug/g (IQR: 38-411) at baseline to 72 ug/g (IQR:3-153) at week 13 (p= 0.01). The CM group saw no change in HBI and there was an increase in FCP by 337 ug/g or 55% from baseline to week 13. The SF-12 yielded two summary scores, the mental component score (MCS-12) and physical component score (PCS-12). The CD-TDI group experienced an increase in both the PCS-12 from 46.9 (SEM: 2.0) to 50.0 (SEM: 1.6) (p=0.10) and the MCS-12 from 49.2 (SEM: 1.8) to 50.9 (SEM: 1.8) (p=0.17), moving both scores to the mean healthy population score of 50, but the changes were not significant. Linear modelling revealed that both FCP (F(1, 58), p<0.001) and CRP (F(1, 62), p<0.001) act as significant predictors of the MCS-12 change score from baseline to week 13. There was no significant change in step count (p=0.41) within the CD-TDI group from baseline to week 13.

**Conclusions:**

It can be concluded that the CD-TDI group experienced a significant decrease in gut inflammation and improved symptoms. Deeper analysis is underway to examine the influence of physical activity parameters on QoL.

**Funding Agencies:**

CIHRNSERC CREATE, University of Calgary

